# SMOC Binds to Pro-EGF, but Does Not Induce Erk Phosphorylation via the EGFR

**DOI:** 10.1371/journal.pone.0154294

**Published:** 2016-04-21

**Authors:** J. Terrig Thomas, Lina Chhuy-Hy, Kristin R. Andrykovich, Malcolm Moos

**Affiliations:** FDA/Center for Biologics Evaluation and Research, Division of Cellular and Gene Therapies Office of Cellular, Tissue, and Gene Therapies, Silver Spring, MD, United States of America; Medical College of Wisconsin, UNITED STATES

## Abstract

In an attempt to identify the cell-associated protein(s) through which SMOC (**S**ecreted **Mo**dular **C**alcium binding protein) induces mitogen-activated protein kinase (MAPK) signaling, the epidermal growth factor receptor (EGFR) became a candidate. However, although in 32D/EGFR cells, the EGFR was phosphorylated in the presence of a commercially available human SMOC-1 (hSMOC-1), only minimal phosphorylation was observed in the presence of *Xenopus* SMOC-1 (*X*SMOC-1) or human SMOC-2. Analysis of the commercial hSMOC-1 product demonstrated the presence of pro-EGF as an impurity. When the pro-EGF was removed, only minimal EGFR activation was observed, indicating that SMOC does not signal primarily through EGFR and its receptor remains unidentified. Investigation of SMOC/pro-EGF binding affinity revealed a strong interaction that does not require the C-terminal extracellular calcium-binding (EC) domain of SMOC or the EGF domain of pro-EGF. SMOC does not appear to potentiate or inhibit MAPK signaling in response to pro-EGF, but the interaction could provide a mechanism for retaining soluble pro-EGF at the cell surface.

## Introduction

The establishment of temporospatial gradients of bone morphogenetic protein (BMP) signaling is important for a diverse array of cellular and systemic processes and requires that the BMP signaling pathway be tightly regulated. Mature BMP proteins can be prevented from binding to their receptors by a number of extracellular antagonists including Noggin, Chordin, Follistatin, Twisted gastrulation, and the DAN (differential screening selected gene abberative in neuroblastoma) family [1]. These BMP antagonists bind directly to BMPs to prevent the ligand-receptor interaction, whereas the transmembrane pseudoreceptor BMP and activin bound protein (BAMBI) associates with the BMP receptor to inhibit activation without direct interaction with BMPs [2].

BMP signaling can also be regulated downstream of the BMP receptor by components of other signaling pathways, including the mitogen-activated protein kinase (MAPK) signaling pathway [3]. Communication between the MAPK pathway and the BMP pathway occurs intracellularly downstream of the BMP receptor. BMPs bind to hetero-tetrameric complexes composed of two type I and two type II serine/threonine kinase receptors. Depending on the type of ligand/receptor interaction, downstream signaling results in the C-terminal phosphorylation of one of two groups of receptor-regulated Smads (R-Smads); Smad 1/5/8 or Smad 2/3. The phosphorylated R-Smads interact with the common mediator Smad4 and translocate into the nucleus to induce transcription of effector genes [4]. The role of MAPK signaling in the regulation of BMP signaling occurs at the level of the R-Smads. The MAPK signaling casacade culminates in the phosphorylation of extracellular signal-regulated kinase (Erk) 1 and 2 [3]. One substrate of diphospho Erk1/2 (dpErk) is the linker region of R-Smads) [3]; phosphorylation of the linker region of R-Smad 1/5/8 by dpErk inhibits BMP signaling by targeting Smad for polyubiquitination and proteasome-mediated degradation [5].

SMOC (**S**ecreted **Mo**dular **C**alcium binding protein) inhibits BMP signaling by inducing MAPK signaling [6]. SMOC, a member of the BM-40/SPARC/Osteonectin family of structurally-related extracellular proteins [7] contains a follistatin-like domain at the N-terminus, two thyroglobulin-like domains separated by a non-homologous domain, and an extracellular calcium (EC) binding domain at the C-terminus [8]. In *Xenopus*, *X*SMOC-1 has a dynamic expression pattern during embryological development, being particularly prominent in the developing brain, eye, and kidney [6]. Functionally, *X*SMOC-1 is required for neurulation [6] in *Xenopus*, and mutations in hSMOC-1 result in autosomal recessive disorders involving limb and eye development [9–11]. Consistent with SMOC inhibiting BMP signaling through the activation of MAPK signaling, SMOC does not affect BMP signaling in the presence of a linker-mutant R-Smad, where the serine Erk phosphorylation sites are substituted for alanine [6]. We showed recently that the domain within SMOC that is required for the induction of MAPK signaling is the N-terminal region containing two thyroglobulin-like type I repeats (submitted work); however, the cell surface receptor by which SMOC induces MAPK signaling remains unknown. Other factors that can inhibit BMP signaling by activating MAPK signaling, such as epidermal growth factor (EGF), fibroblast growth factor (FGF), and insulin-like growth factor (IGF) all signal by binding to receptor tyrosine kinases (RTKs) [3, 12]. In an attempt to identify potential cell surface receptor(s) that are activated by SMOC we conducted a phosphoproteomic screen of HEK293 cells following exposure to *X*SMOC-1 protein.

## Materials and Methods

### Recombinant SMOC Proteins

Full length *Xenopus X*SMOC-1, *X*SMOC-1ΔEC (25 to K309) lacking the EC domain, and *X*SMOC-1EC containing the EC domain only (K309 to end) in the pET- 28b(+) vector (Novagen) were expressed in the Shuffle^®^T7 Express E.coli strain C3029 (New England Biolabs). Refolding was based on a previously described protocol [13] with modifications. A detailed protocol will be published elsewhere; a brief description is presented here. Following bacterial cell lysis solubilized inclusion bodies were applied to and eluted from Ni-NTA agarose (Qiagen). Proteins were refolded by rapid dilution into 100mM Tris/HCl, pH 9.0, 600mM L-Arginine, 6mM reduced L-Glutathione, 0.6mM oxidized L-Glutathione, and 2mM CaCl_2_. Soluble refolded proteins were dialyzed against 20mM Tris/HCl pH 7.5, 300mM NaCl, 2mM CaCl_2_, concentrated, and separated by size exclusion chromatography on Superdex-200 (GE Healthcare). Fractions containing major peaks were pooled and concentrated.

### Cell Culture

HEK293 cells (ATCC^®^ CRL-1573^™^), which are known to respond to BMPs, were cultured in DMEM medium supplemented with 10% fetal bovine serum (FBS). The murine myeloid progenitor cell line 32D and 32D cells stably transfected with the EGF receptor (32D/EGFR), kindly provided by Gibbes Johnson [14], were cultured in RPMI 1640 medium supplemented with 10% FBS and 5% medium conditioned by WEHI-3B cells [14]. Prior to the addition of recombinant proteins cells were serum-starved in their respective serum-free media for one hour. The recombinant proteins used were *X*SMOC-1, *X*SMOC-1ΔEC, *X*SMOC-1EC, expressed in bacteria and refolded, or human SMOC-1 (R&D Systems #6074-SM) derived from a mammalian host-vector system.

### Immunoblotting

Cell lysates were prepared by extraction in 6M Urea, 25mM Tris base, 2% SDS; aliquots (10μg) were mixed with 1X LDS sample buffer (Invitrogen)/2% mercaptoethanol prior to analysis by SDS-PAGE using Novex 10% Nu-PAGE gels (Invitrogen) and the MES buffer system. Immunoblot analyses were performed using the Novex XCell SureLock^®^ Mini-Cell system (Life Technologies) and nitrocellulose membranes (Invitrogen). Transferred proteins were detected using IRDye-labeled secondary antibodies and the Odyssey infrared imaging system (Li-COR Biosciences). The primary antibodies used were phospho p44/42 MAPK (dpErk), p44/42 MAPK, phospho-MEK1/2, EGFR (Cell Signaling Technology #9101, 9107, 9121, and 2239), pEGFR (Y1172; Abcam^®^ #ab47364), mature EGF (EMD Millipore # PC08), and pro-EGF (R and D Systems #AF4289).

### Immunoprecipitation/Immunodepletion

For immunoprecipitation experiments, 10μg of peptide antibodies specific to *X*SMOC-1ΔEC (SDRDRDPQCNPHCTRPQHK) or *X*SMOC-1EC (GSFPPGKRPGSNPFSR) produced in rabbits (Biomatik, Canada) were incubated overnight at 4°C in 500μl Tris-Buffered Saline, pH.7.5 with 0.05% Tween 20 (TBST) and 5μg of pro-EGF (R&D Systems #4289-EG/CF) plus BSA (100μg) with or without 5μg of recombinant *X*SMOC-1, *X*SMOC-1ΔEC, or *X*SMOC-1EC. The proteins were subsequently incubated with pre-washed Pierce^®^ Protein A/G magnetic beads (Thermo Scientific) for 1hr at room temperature followed by washing with TBST/0.5M NaCl/0.1% SDS to remove proteins bound non-specifically. Proteins were eluted from the beads in 1X LDS sample buffer (Invitrogen)/2% mercaptoethanol at 75°C for 10min. For immunodepletion experiments, a pro-EGF antibody (R and D Systems #AF4289) was coupled directly to magnetic Dynabeads^®^ (Life Technologies) and 50μg hSMOC-1 (R and D Systems) in 500μl DMEM applied. The beads were washed with DMEM and the washes kept for subsequent analysis before elution with 50mM glycine pH 2.7–3.0.

### Heparin-sepharose binding

For SMOC/pro-EGF/EGF heparin-binding studies, 5μg of *X*SMOC-1 and pro-EGF in 50μL of 1x PBS/0.5M NaCl, pH7.4 were added to 2μL of pre-equilibrated heparin Sepharose (HS) high performance beads (GE Life Sciences) and mixed with rotation for 15 minutes at room temperature. The beads were centrifuged (350 x g for 2 minutes) and the supernatant removed. The protein-heparin bead mixture was washed twice with 1X PBS/0.5M NaCl (wash buffer) before suspension in wash buffer containing 5μg pro-EGF and incubation for a further 15 minutes. The mixture was centrifuged, washed twice in wash buffer before elution with 40μl of 1X LDS sample buffer (Invitrogen)/0.1M DTT for 5 minutes at 95°C. Pro-EGF (5μg) heparin- binding studies were conducted in the presence of HS (2μl) pre-equilibrated in 5mM imidazole pH.7.0 [15] and 90mM, 154mM, or 500mM NaCl. After 15 minutes the pro-EGF/HS bead mixture was washed three times in the respective buffers and eluted in 40μl of 1xLDS sample buffer. All supernatants were analyzed on a 10% NuPAGE gel and visualized by Coomassie staining.

### *Xenopus* Whole Mount Hybridization *In situ*

Frogs (*Xenopus laevis*), purchased from NASCO (Fort Atkinson, WI), were housed and maintained in aquaria approved by the FDA White Oak Campus Animal Care and Use Committee (ACUC). Prior to testes collection, male frogs were euthanized by anesthesia in a 2% solution of tricaine methane-sulphonate, a protocol approved by the ACUC. Frog embryos were manipulated using standard methods [16, 17] and euthanized by anesthesia when the required developmental stage was reached (the study was approved by the ACUC). For whole mount hybridization *in situ*, *Xenopus* embryos (stage 26) were transferred to medium sized baskets for automated hybridization in situ using an InsituPro VSi instrument (Intavis Bioanalytical Instruments) programed to emulate the manual method described previously [6]. A 1090bp cDNA fragment for *Xenopus laevis* pro-EGF was obtained by RT-PCR using the forward primer 5’-TGGAATCATGGCTGTACTCTTGG-3’, reverse primer 5’-GCATGTTGCCTCGAAGACGTAC-3’, and total RNA from stage 20 *Xenopus* embryos as template. cRNA probes for pro-EGF and *X*SMOC-1 [6] were produced using MEGAscript T3 or T7 *in vitro* transcription kits (Ambion), incorporating digoxigenin-UTP. For colorimetric detection, signals were developed using an alkaline phosphatase-conjugated antibody to digoxigenin and BM-Purple (Roche Applied Science). Darkfield images were captured using an Olympus SZX16 stereo microscope with LED ring illumination and cellSens Dimension software (v1.12).

### Proximity Ligation Assay (PLA)

Prior to conducting the PLA, HEK293 cells were shown to express pro-EGF by RT-PCR using the forward primer 5’-ATGAGCAATTGGTGGTGGATGCTG-3’ and reverse primer 5’-TAAAGGCTTCCAGCCACCTCTGAA-3’. HEK293 cells were seeded onto 8-well chamber slides (Millicell^®^ EZ slides, Millipore) at 3 x 10^4^ cells/well and cultured for 24hrs in DMEM supplemented with 10% FBS. The medium was removed and replaced with serum-free medium for 1hr followed by incubation in serum-free medium containing *X*SMOC-1 (100μg/ml) for 5 min. The wells were rinsed in PBS, fixed in 4% paraformaldehyde for 15 min and permeabilized in PBS/0.5% Triton-X-100 for 10 min. The chambers were removed and the PLA was performed on the slides according to the manufacturer’s instructions for Duolink in situ Red detection (Olink Biosciences). After blocking for 30 min at 37°C, the slides were incubated for 50 min at 37°C with primary antibodies raised in rabbit (*X*SMOC-1) or goat (pro-EGF; R&D Systems #AF4289) diluted 1/500in Duolink diluent. After washing, SMOC/pro-EGF complexes were detected using rabbit PLUS and goat MINUS secondary PLA antibodies prepared and used according to the manufacturer’s instructions. Following a second wash step, the ligation and amplification reactions for detection of the red fluorophore were carried as described by the manufacturer. The slides were washed, counterstained with DAPI, and imaged by confocal microscopy (Zeiss LSM710).

## Results

### Induction of MAPK Signaling by SMOC

**Fig 1:**
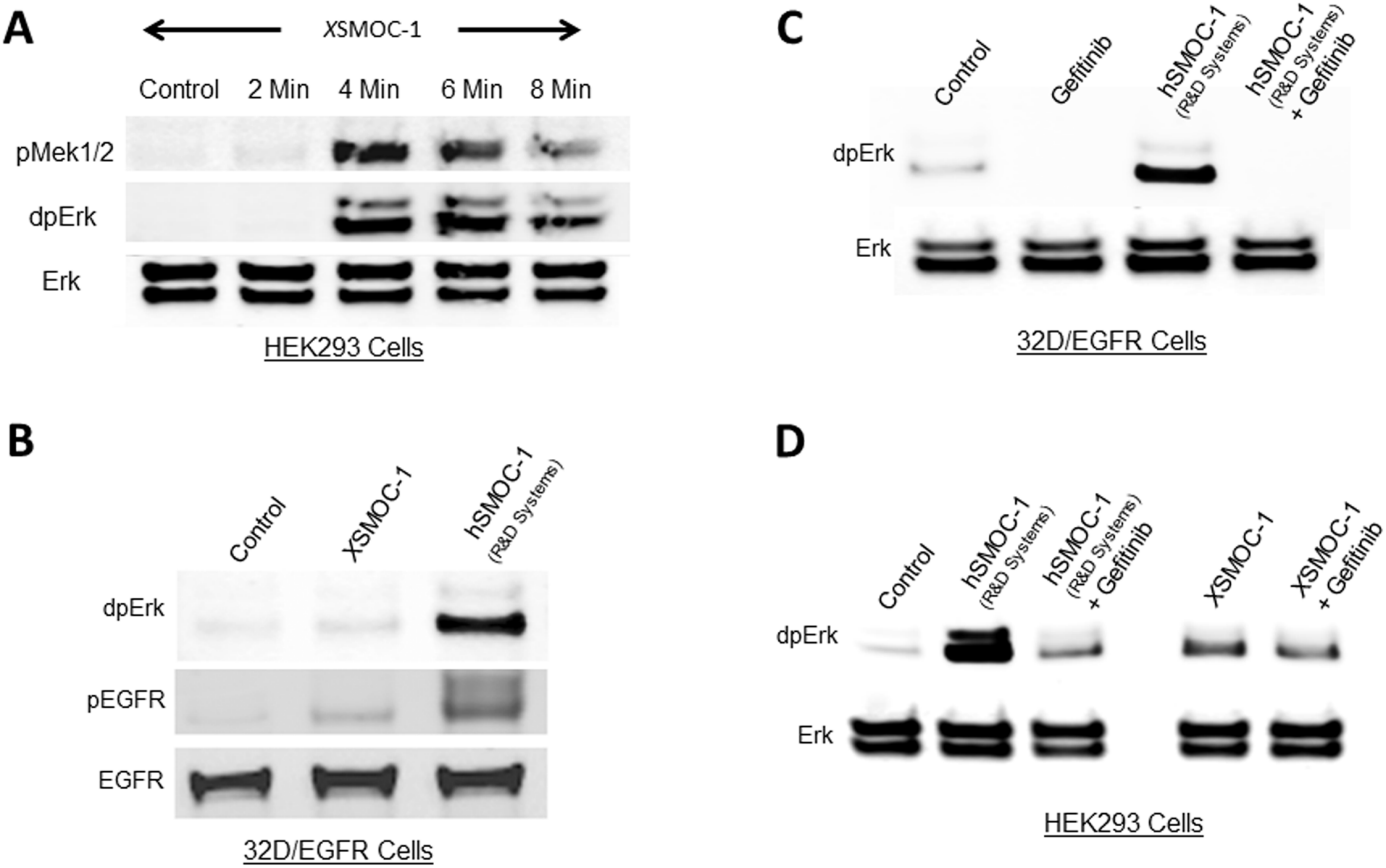
Induction of MAPK signaling by SMOC does not require the EGF receptor. Immunoblot analysis of serum-starved HEK293 (A, D) or 32D/EGFR (B, C) cell lysates following addition of *X*SMOC-1 (100μg/ml) or hSMOC-1 (50μg/ml). (A) Following addition of *X*SMOC-1 to HEK293 cells, phosphorylation of MEK1/2 and Erk was evident at four minutes. (B) Phosphorylation of Erk and EGFR (Y1172) in 32D/EGFR cells was weak in the presence of *X*SMOC-1 compared to hSMOC-1. (C) Phosphorylation of Erk by hSMOC-1 in 32D/EGFR cells was blocked by the small molecule EGFR inhibitor, Gefitinib (5μM). (D) Phosphorylation of Erk by SMOC in HEK293 cells does not require the EGFR; *X*SMOC-1 and hSMOC-1 continue to induce Erk phosphorylation in the presence of Gefitinib but, in the absence of Gefitinib, hSMOC-1 stimulated ERK phosphorylation more than *X*SMOC-1. Non-phosphorylated Erk and EGFR are shown as loading controls. The results presented are representative of experiments conducted at least three times.

We showed previously that overexpression of *X*SMOC-1 mRNA by injection of *Xenopus* embryos at the two cell stage resulted in an increase of phosphorylation of Erk-1/2 (dpErk) in ectodermal explants (animal caps) removed at stage 9 and incubated to stage 21[6]. To determine the temporal relationship between SMOC and the induction of MAPK signaling we used *X*SMOC-1 expressed in bacteria and refolded. When *X*SMOC-1 (100μg/ml) was added to serum-starved human HEK293 cells, the mitogen-activated protein kinase kinase (MAPKK) MEK 1/2 and Erk 1/2 (dpERK) were phosphorylated within four minutes (Fig 1A). In an attempt to identify upstream signaling events, a high throughput phosphorylation screen (Kinex^™^ Antibody Microarray, Kinexus Bioinformatics Corp) was conducted on HEK293 cell lysates following a six minute exposure to *X*SMOC-1. As expected, phosphorylation of both MEK 1/2 and Erk 1/2 were significantly elevated, providing some verification that the screen should be informative (not shown). Of the membrane-associated receptors represented in the screen, the epidermal growth factor receptor (EGFR) showed increased phosphorylation in response to *X*SMOC-1. However, a subsequent immunoblot validation study (Kinetworks^™^) using three different pEGFR antibodies (Y1069, Y1110, and Y1138) was negative (not shown).

As it was plausible that SMOC may activate the EGFR at alternative phosphorylation sites, we nevertheless tested whether SMOC could activate MAPK signaling via the EGFR. For these studies we used the murine 32D myeloblast-like cell line, which does not express the EGFR, and 32D cells stably transfected with the EGFR [14]. When *X*SMOC-1 was added to 32D cells, neither EGFR or ERK were phosphorylated (not shown); when it was added to 32D/EGFR cells, both EGFR (Y1172) and ERK displayed only weak phosphorylation (Fig 1B, lane 2). In contrast, the addition of recombinant human SMOC-1 (hSMOC-1), which was expressed in murine cell line, promoted much greater levels of pEGFR and dpERK compared to *X*SMOC-1, expressed in bacteria (Fig 1B, lane 3). While the result was puzzling, we proceeded to assess whether the EGFR contributes directly to the induction of MAPK signaling by SMOC using the EGFR small molecule inhibitor, Gefitinib. When Gefitinib (5μM) was added to 32D/EGFR cells, phosphorylation of ERK in response to hSMOC-1 was abolished (Fig 1C), indicating effective inhibition of the EGFR. However when Gefitinib was added to HEK293 cells, while MAPK signaling in the presence of hSMOC-1 was reduced, it was still elevated relative to control (Fig 1D). Furthermore, Gefitinib had no effect on MAPK signaling in the presence of *X*SMOC-1 (Fig 1D), indicating that bacterially expressed *X*SMOC-1 does not signal primarily through the EGFR in HEK293 cells.

### SMOC binds Pro-EGF, but does not signal through the EGFR

**Fig 2:**
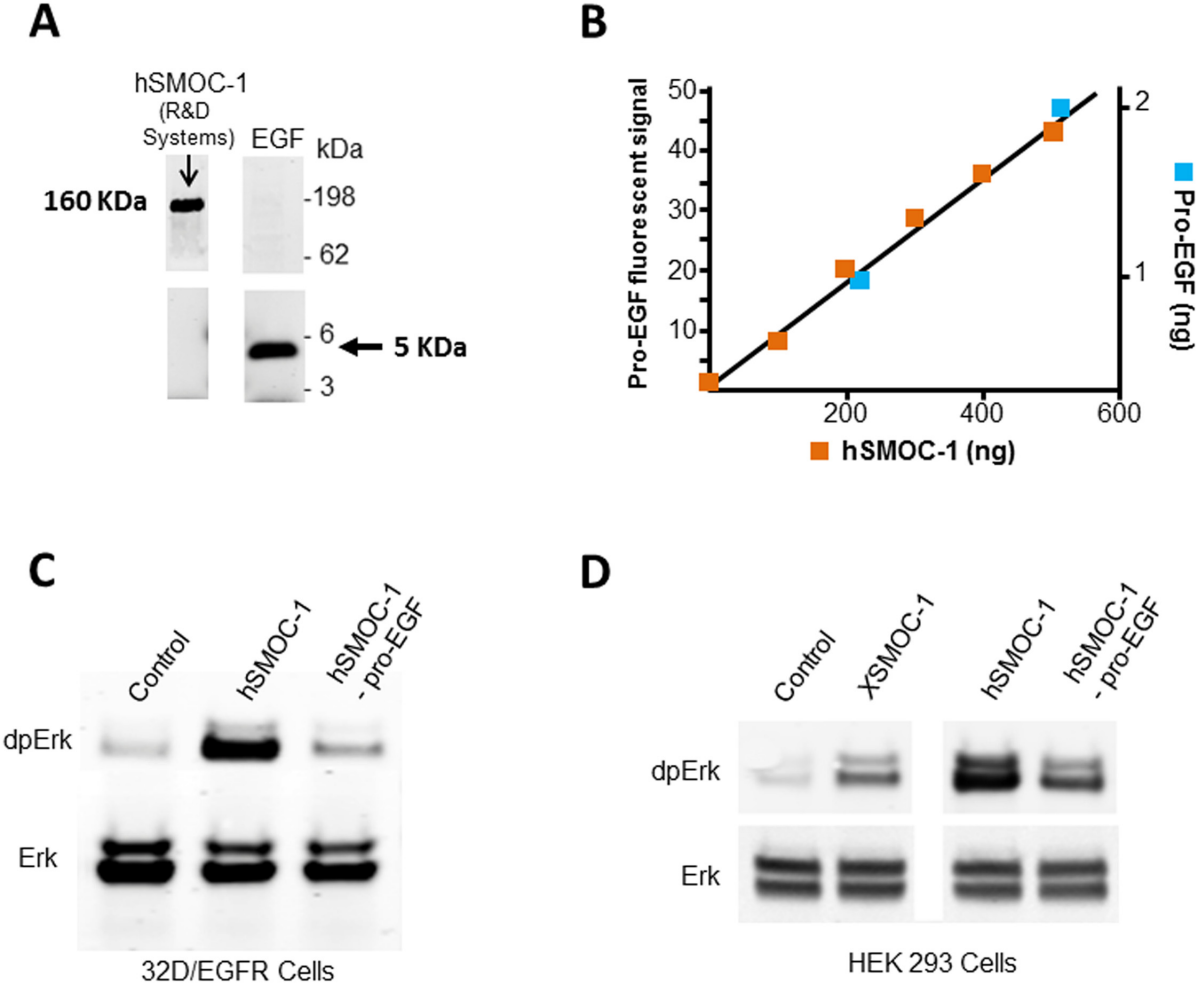
Pro-EGF is present as an impurity in a commercial hSMOC-1 product. (A) Immunoblot of 5μg hSMOC-1 (R&D systems #6074-SM) with an antibody to mature EGF (EMD Millipore #PC08). The band detected at approximately 160 kDa is consistent with the mass of pro-EGF. Mature EGF (50ng), detected at 5kDa, is shown as control. (B) Immuno-quantitation of pro-EGF in hSMOC-1. Fluorescence scan analysis of pro-EGF signals obtained following immunoblotting of hSMOC-1 (R&D Systems) and recombinant pro-EGF (R&D Systems #4289-EG) at the amounts shown. The left-hand Y axis displays the pro-EGF fluorescence signal intensity; the X axis displays the amounts of commercial hSMOC-1 analyzed (blue squares); the right hand Y axis displays the known amounts of recombinant pro-EGF analyzed (red squares). Note: Additional pro-EGF data points were used for the best fit analysis, but the graph was cropped to show the intersect point with hSMOC-1 more clearly. (C) Immunoblot of 32D/EGFR cell lysates showing increased phosphorylation of Erk following a six minute exposure to hSMOC-1 or hSMOC-1 immuno-depleted of pro-EGF (hSMOC-1 –proEGF). (D) Immunoblot of HEK293 cell lysates showing Erk phosphorylation following a six minute exposure to *X*SMOC-1, hSMOC-1, or hSMOC-1 –proEGF; the apparent higher potency of undepleted hSMOC-1 was presumably due to the pro-EGF impurity. The cell culture experiments were conducted in triplicate and the results presented are representative of those obtained.

The finding that hSMOC-1 induced MAPK signaling in 32D/EGFR cells more effectively than *X*SMOC-1 was examined further. Based on the information provided by the manufacturer, the hSMOC-1 (R&D Systems) was expressed and purified from a murine myeloma cell line. While it was possible that post-translational modifications to the hSMOC-1 protein may account for the different activities, we first assessed whether the hSMOC-1 product contained any EGF as an impurity from the tissue culture medium. Whereas no mature EGF protein could be detected by immunoblot analysis of the hSMOC-1 (<1ng/50μg hSMOC-1; not shown), an immunoreactive band was apparent at approximately 160kDa, the expected molecular mass of pro-EGF (Fig 2A). Following SDS-PAGE, Coomassie-stained bands in the 160kDa region were excised and submitted for in-gel tryptic digest followed by sequencing by MS-MS (ITSI Biosciences). Three of the peptide sequences obtained had 100% identity to human pro-EGF (Table 1), confirming the presence of pro-EGF as an impurity in the commercial hSMOC-1.

**Table 1:** Human pro-EGF peptide sequences obtained from MS-MS sequencing (ITSI Biosciences) of human SMOC-1 (R&D Systems).

Species	Peptide sequence	#PSMs	Human Pro-EGF (# P01133)
Human	**LIEEGVDVPEGLAVDWIGR**	1	L549 to R567
Bovine	**LIEE***E***V***GL***PEGLAVDWIGR**		
Murine	**LI***T***EGVD***TL***EGLALDWIGR**		
Human	**LFWTDTGINPR**	2	L612 to R622
Bovine	**LFWTD***I***GINPR**		
Murine	**LFWTD***V***G***MS***PR**		
Human	**TCLALDGHQLLAGGEVDLK**	1	T779 to K797
Bovine	*M***CLAL***H***GH***RI***L***SDNMTNCS*		
Murine	*M***CL***PQ***D***-YPI***L***S***G***ENA***DL***S*		

The three human pro-EGF peptide sequences obtained following excision from SDS-PAGE gels in the 160 kDa region are shown aligned to homologous bovine and mouse peptides. The number of peptide spectral matches (PSM) for each peptide is indicated and the normalized spectral abundance factor for pro-EGF was calculated to be 0.008106. A complete list of proteins identified from the MS-MS analysis with PSMs ≥2 is provided (S1 Table).

We next determined whether pro-EGF was present in other recombinant proteins produced by R&D Systems using this cell line; immunoblot analyses of human noggin (#6057-NG-025) and BMP6 (#507-BP-020/CF) were negative for pro-EGF (not shown), demonstrating that pro-EGF is not a common impurity in recombinant proteins expressed in the murine myeloma cell line. To determine the amount of pro-EGF in the hSMOC-1 product we conducted a quantitative immunoblot analysis; a dilution series of known amounts of hSMOC-1 and recombinant pro-EGF were analyzed using an antibody specific to pro-EGF detected with a fluorescent IRDye 680 secondary antibody (Licor Biosciences). Based on scanning (Odessey^®^ Image System) of fluorescent signal intensities (Fig 2B), pro-EGF was present at approximately 5ng/μg of hSMOC-1 (i.e., 0.5%).

We tested whether the pro-EGF impurity in the commercial hSMOC-1 product was sufficient to initiate MAPK signaling in 32D/EGFR cells by removing the pro-EGF using an anti-pro-EGF antibody coupled to epoxy-activated Dynabeads^®^. When hSMOC-1 immunodepleted of pro-EGF was added to 32D/EGFR cells, only a small increase in dpErk was observed (Fig 2C), similar to that observed with *X*SMOC-1 (Fig 1B). Furthermore, addition of pro-EGF-depleted hSMOC-1 to HEK293 cells resulted in induction MAPK signaling (Fig 2D), but at a reduced level compared to the untreated hSMOC-1. This indicated that the increased potency of the hSMOC-1, compared to *X*SMOC-1 (Fig 1B), was most likely due to the presence of pro-EGF in the commercial preparation. Indeed, human SMOC-2 (R&D Systems #5140-SM), which was produced using Chinese Hamster Ovary (CHO) cells, did not contain detectable levels of pro-EGF and did not markedly induce MAPK signaling in 32D/EGFR cells (not shown).

**Fig 3:**
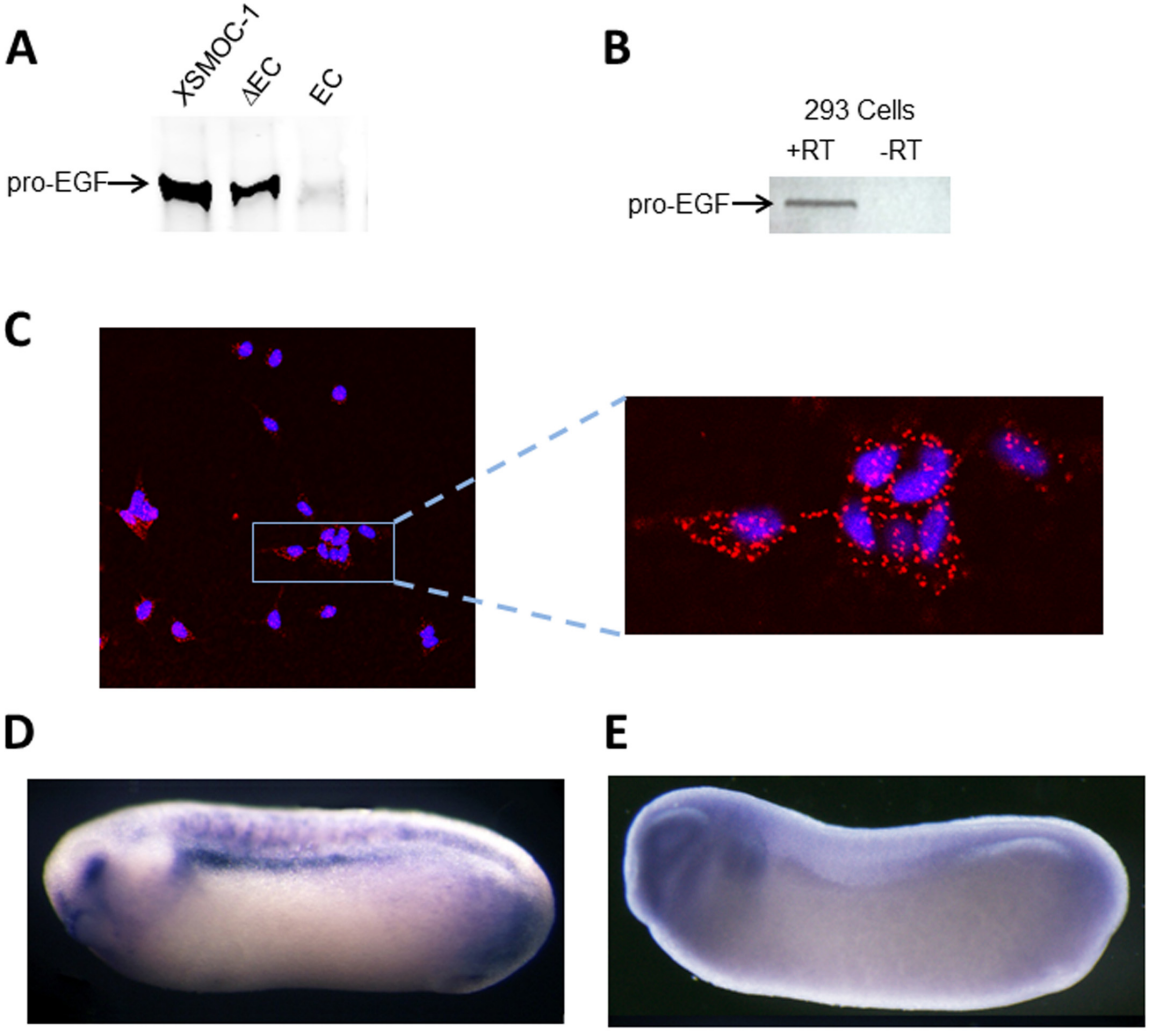
*X*SMOC-1 binds to pro-EGF and co-localizes with pro-EGF *in vivo*. (A) Immunoblot of pro-EGF following co-immunoprecipitation of pro-EGF with *X*SMOC-1, *X*SMOC-1 ΔEC, or *X*SMOC-1 EC in the presence of TBST/0.1% SDS; pro-EGF binds to *X*SMOC-1 and *X*SMOC-1 ΔEC, but not *X*SMOC-1EC. (B) RT-PCR analysis of HEK293 cells showing positive signal for pro-EGF(C) Representative confocal image showing co-localization of *X*SMOC-1 and pro-EGF (red fluorophore) on HEK293 cells using the PLA method. Nuclei are stained blue with DAPI. (D, E) Representative whole mount hybridization *in situ* images of *Xenopus* neurula embryos (stage 26) stained for *X*SMOC-1 (C) or pro-EGF (D). The locations of the eye (e) and pronephros (pn) are indicated.

Since pro-EGF was such an unexpected impurity, not present in other recombinant proteins expressed in the same cell line, we studied whether SMOC is able to bind to pro-EGF. In co-immunoprecipitation experiments, using an *X*SMOC-1 antibody coupled to Protein A/G magnetic beads, we found that *X*SMOC-1 and pro-EGF form a stable complex, remaining bound even in the presence of 0.5M NaCl/0.1% SDS (Fig 3A). By using SMOC antibodies specific to either *X*SMOC-1ΔEC or *X*SMOC-1EC, we found that *X*SMOC-1 and *X*SMOC-1ΔEC co-precipitated with pro-EGF, whereas *X*SMOC-1EC did not (Fig 3A). To test whether SMOC could interact with pro-EGF at the cellular level, we used the Proximity Ligation Assay (PLA), where a positive signal is obtained only if the epitopes for antibodies to each protein are within 40 nm of each other [18]. HEK293 cells, which were shown to express pro-EGF by RT-PCR, (Fig 3B) were used for this assay. After adding *X*SMOC-1 to the culture media for six minutes, the cells were washed prior to conducting the PLA. The primary antibodies used for the PLA were a SMOC antibody raised in rabbit and a pro-EGF antibody raised in goat. SMOC/pro-EGF complexes were detected using rabbit PLUS (+) and goat MINUS (-) oligonucleotide-modified secondary antibodies (PLA probes). Following ligation of two additional oligonucleotides, if the PLA probes were in close proximity a circular DNA would be formed and, following amplification, visualized with a red fluorophore. The positive signal obtained for the SMOC/pro-EGF PLA (Fig 3C), indicated that SMOC co-localized with pro-EGF on the cell membrane in this assay. No signal was obtained under control conditions where either SMOC was not added to the HEK293 cells or where the secondary oligo-modified antibodies were both PLUS or both MINUS (not shown). To determine whether the two genes are co-expressed *in vivo*, whole mount hybridization *in situ* was conducted on *Xenopus* embryos at the neurula stage of development, a stage at which SMOC expression is pronounced, predominantly in the ventral aspect of the developing eye, the mid- and hind-brain, and the pronephros ([6], Fig 3D). Examination of pro-EGF expression at this stage demonstrated it to be expressed throughout the head region, the eye vesicle, branchial arches, and pronephros (Fig 3E).

### SMOC bound to heparin sepharose can also bind to pro-EGF

**Fig 4:**
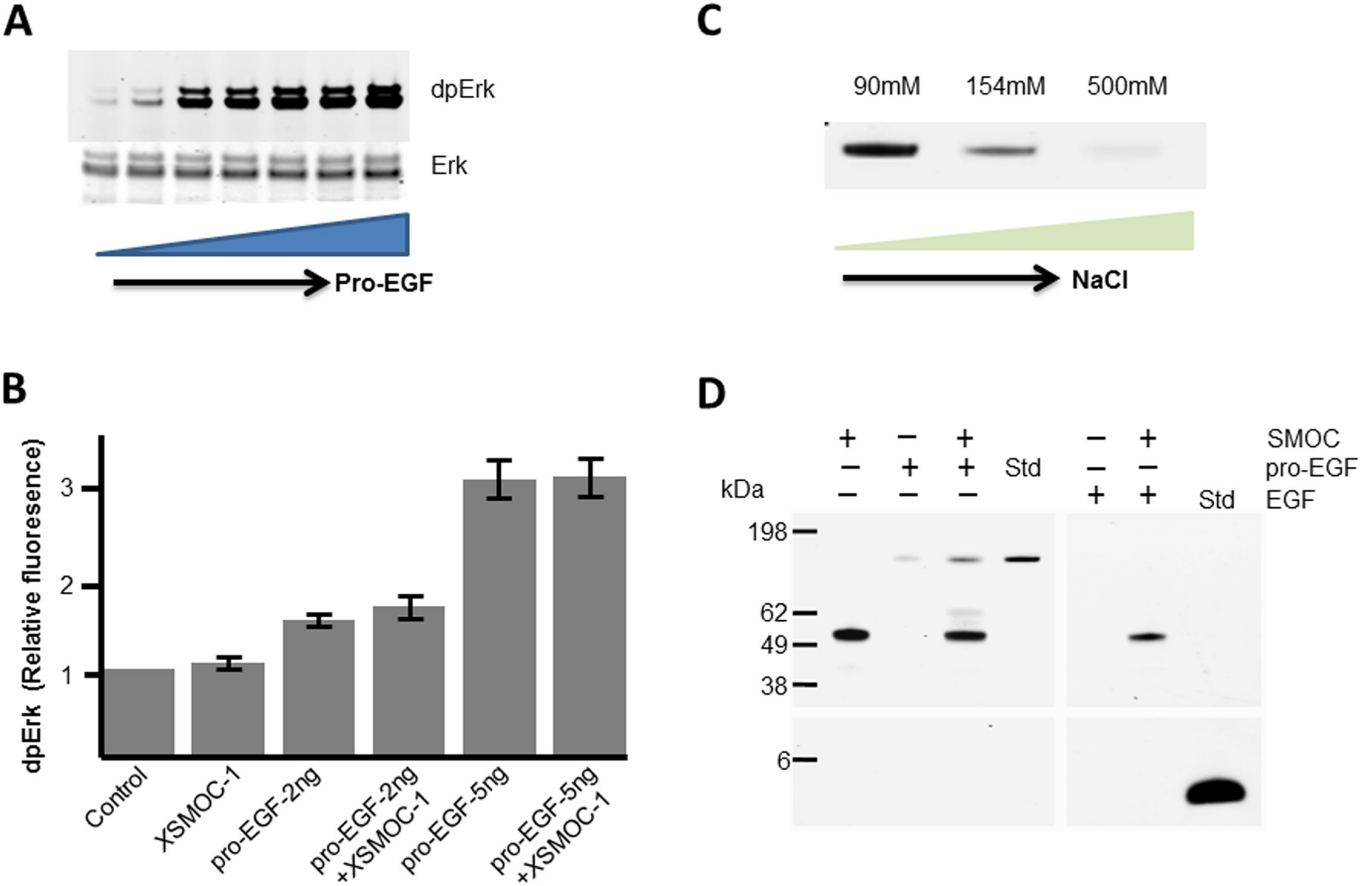
*X*SMOC-1 does not potentiate MAPK signaling by pro-EGF, but can bind to pro-EGF when bound to heparin sepharose. (A) Immunoblot of 32D/EGFR cell lysates showing Erk phosphorylation (dpErk) following a six minute exposure to a dilution series (0–500ng/ml) of pro-EGF. Total Erk is shown as loading control. (B) Graph showing relative dpErk fluorescence obtained on immunoblots from triplicate experiments of 32D/EGFR cells following a six minute exposure to submaximal concentrations of pro-EGF (2ng or 5ng/ml) in the presence or absence of *X*SMOC-1 (100μg/ml).The level of dpErk by pro-EGF was not significantly affected by *X*SMOC-1. (C) Coomassie-stained SDS-PAGE showing the heparin sepharose (HS) elution profile of pro-EGF in the presence of increasing concentrations of NaCl (D) Coomasie-stained SDS-PAGE showing HS elution profiles (±) following incubation of 5μg *X*SMOC-1 with 5μg of either pro-EGF or EGF in PBS/500mM NaCl, compared to each protein alone. A standard (std) lane is provided for pro-EGF and EGF to demonstrate their expected migration position.

Having established that SMOC and pro-EGF can bind to each other and could potentially be co-expressed, we examined whether *X*SMOC-1 affects the induction of MAPK signaling by pro-EGF. For this study we first determined a submaximal concentration of pro-EGF for inducing MAPK signaling. When pro-EGF was added to 32D/EGFR cells in a dilution series, Erk phosphorylation was found to be submaximal between 2ng/ml and 5ng/ml, and maximal at 10ng/ml (Fig 4A). To assess the effect of SMOC on the activity of pro-EGF, *X*SMOC-1 (50μg/ml) was added to 32D/EGFR cells together with submaximal concentrations of pro-EGF (2ng/ml or 5ng/ml). Analysis of dpErk showed that *X*SMOC-1 had no apparent effect on the level of MAPK signaling in the presence of submaximal amounts of pro-EGF (Fig 4B). As SMOC can bind heparan sulfate proteoglycans (HSPGs), another possible function of the SMOC/pro-EGF interaction would be to restrict diffusion of pro-EGF once it has been cleaved from the cell membrane. This would require that SMOC can bind to pro-EGF and HSPGs simultaneously. As it has been reported that pro-EGF has some affinity for heparin [15], we first examined this interaction. As reported previously [15], pro-EGF binds to heparin sepharose (HS) at 90 mM NaCl (Fig 4C). However, we found the binding affinity to be relatively weak; pro-EGF was only partially bound at 154 mM NaCl, the physiological concentration in PBS, and did not bind at 500mM NaCl (Fig 4C). In contrast, SMOC remained bound to HS at 500mM NaCl (Fig 4D). Under these conditions, pro-EGF bound to SMOC when SMOC was bound to HS, whereas EGF showed no affinity for either HS or SMOC (Fig 4D).

## Discussion

We showed previously that MAPK signaling was induced in *Xenopus* ectodermal explants following overexpression of *X*SMOC-1 mRNA [6]. However, while this assay was informative, it did not provide any information as to the temporal nature of the effect. Using pure recombinant SMOC protein allowed us to conduct a short-duration time course experiment in HEK293 cells demonstrating that Erk phosphorylation was maximal at six minutes. Consequently, during this time, SMOC must bind to one or more putative cell associated protein(s) to initiate the downstream phosphorylation events of MAPK signaling. Analysis of the results of a phospho-antibody array screen, following a six minute exposure of HEK293 cells to *X*SMOC-1, suggested the epidermal growth factor receptor (EGFR) as a potential candidate. Examination of the role of the EGFR in promoting MAPK signaling in response to SMOC, using 32D/EGFR cells, revealed the following: MAPK signaling was strongly induced by a commercially available hSMOC-1, but only weakly induced by recombinant *X*SMOC-1 produced in bacteria and refolded. The difference was explained by the presence of pro-EGF as an impurity in the hSMOC-1 product; hSMOC-1 immunodepleted of pro-EGF induced Erk phosphorylation only weakly. Conversely, in HEK293 cells, addition of either *X*SMOC-1 or hSMOC-1 depleted of pro-EGF induced Erk phosphorylation strongly. Furthermore, the addition of the EGFR inhibitor Gefitinib to HEK293 cells did not affect the ability of SMOC to induce Erk phosphorylation, suggesting that SMOC does not signal primarily through the EGFR in these cells. While the cell associated protein(s) required for the induction of MAPK signaling by SMOC remain(s), unknown, we now know that the necessary signaling apparatus is present in HEK293 cells, but not 32D cells. Future analysis of the transcriptomes of these two cell lines may lead to the identification of potential cell-associated protein(s) required for the induction of MAPK signaling by SMOC.

How pro-EGF was present in the hSMOC-1 product is not known. According to the manufacturer, the cells used to produce hSMOC-1 are cultured in the presence of bovine serum. This would be the most likely source, as serum is known to contain pro-EGF [19]. However, immunoanalysis failed to detect pro-EGF in other proteins produced from the same murine myeloma cell line or the highly similar human SMOC-2, produced in a different cell line (CHO). In addition, the pro-EGF peptides identified in hSMOC-1 by mass spectroscopy were human in origin. Consequently, it is unlikely that the pro-EGF present in hSMOC-1 was derived from the bovine serum used for cell culture. While confusing, our data suggest that the presence of pro-EGF may result from a direct interaction with SMOC. Immunoprecipitation assays showed that both *X*SMOC-1 and *X*SMOC-1ΔEC can bind to pro-EGF quite tightly, whereas *X*SMOC-1EC domain does not. However, the estimation that pro-EGF constitutes only approximately five nanograms per microgram of hSMOC-1 is consistent with our finding that the potency of hSMOC-1, depleted of the SMOC/pro-EGF complex, is not measurably affected in HEK293 cells. A preliminary analysis of the region within pro-EGF that binds to SMOC suggests that the EGF domain is not involved as SMOC did not bind to mature EGF. Consequently, as the remainder of the pro-EGF molecule contains eight additional EGF-like domains and eight LDL-receptor class B domains [20], it is most probable that SMOC binds to one or more of the LDL-receptor class B domains.

Whether SMOC interacts with pro-EGF *in vivo* is not known, but preliminary results from hybridization *in situ* would suggest that this is possible; pro-EGF has a similar expression pattern to that of *X*SMOC-1 in the head and pronephros regions of neurula stage *Xenopus* embryos. While beyond the scope of this investigation, a more detailed co-expression analysis at the protein level may highlight particular locations and/or stages of development that are most compelling for further study. However, whereas co-localization is possible *in vivo*, the biological significance of the SMOC/pro-EGF interaction is unclear; binding of *X*SMOC-1 to pro-EGF in 32D/EGFR cells did not appear to enhance or inhibit the ability of pro-EGF to induce MAPK signaling via the EGFR. Based on previous studies showing that SMOC and the *Drosophila* orthologue, Pentagone, bind to cell-associated HSPGs [21],[22], a potential function of the interaction could be that SMOC acts to restrict diffusion of pro-EGF following cleavage from the cell membrane. Pro-EGF has only a low affinity for heparin-sepharose at physiologic salt concentrations, which would limit any association of pro-EGF with HSPGs at the cell surface or within the extracellular matrix. Our data indicate that heparin-bound SMOC can bind to pro-EGF with high affinity, suggesting that SMOC bound to HSPGs *in vivo* could bind pro-EGF and prevent further diffusion. The indirect binding of pro-EGF to HSPGs via SMOC would constitute a similarity to the EGF family members heparin-binding EGF (HB-EGF) [23], amphiregulin [24], betacellulin [25], and neuregulin [26]. These proteins contain N-terminal HB sites in their pro-domains, which have been shown for to play an important role in restricting HB-EGF and neuregulin diffusion following proteolytic release [26–28].

In conclusion, we have identified a new characteristic of SMOC; in addition to being a BMP antagonist, SMOC is able to bind to soluble pro-EGF. The interaction does not require the SMOC EC domain and likely involves the binding of SMOC to one or more of the LDL-R class B repeats within pro-EGF. SMOC does not appear to potentiate or inhibit the ability of pro-EGF to induce MAPK signaling, but could function to retain pro-EGF at the cell membrane through binding to both pro-EGF and HSPGs.

## Supporting Information

S1 Table Protein identification summary of all peptides identified by MS/MS analysis (ITSI Biosciences) of human SMOC-1. (XLSX)
